# Optimization of training periods for the estimation model of three-dimensional target positions using an external respiratory surrogate

**DOI:** 10.1186/s13014-018-1019-9

**Published:** 2018-04-19

**Authors:** Hiraku Iramina, Mitsuhiro Nakamura, Yusuke Iizuka, Takamasa Mitsuyoshi, Yukinori Matsuo, Takashi Mizowaki, Ikuo Kanno

**Affiliations:** 1 Department of Nuclear Engineering, Graduate School of Engineering, Kyoto University, Nishikyo-ku, Kyoto, 615-8530 Japan; 2 Department of Radiation Oncology and Image-applied Therapy, Graduate School of Medicine, Kyoto University, 54 Kawahara-cho, Shogoin, Sakyo-ku, Kyoto, 606-8507 Japan; 3 Division of Medical Physics, Department of Information Technology and Medical Engineering, Human Health Sciences, Graduate School of Medicine, Kyoto University, 53 Kawahara-cho, Shogoin, Sakyo-ku, Kyoto, 606-8507 Japan

**Keywords:** 3D target motion estimation, Baseline drift correction period, CBCT projection, External surrogate, Training period

## Abstract

**Background:**

During therapeutic beam irradiation, an unvisualized three-dimensional (3D) target position should be estimated using an external surrogate with an estimation model. Training periods for the developed model with no additional imaging during beam irradiation were optimized using clinical data.

**Methods:**

Dual-source 4D-CBCT projection data for 20 lung cancer patients were used for validation. Each patient underwent one to three scans. The actual target positions of each scan were equally divided into two equal parts: one for the modeling and the other for the validating session. A quadratic target position estimation equation was constructed during the modeling session. Various training periods for the session—i.e., modeling periods (*T*_M_)—were employed: *T*_M_ ∈ {5,10,15,25,35} [s]. First, the equation was used to estimate target positions in the validating session of the same scan (intra-scan estimations). Second, the equation was then used to estimate target positions in the validating session of another temporally different scan (inter-scan estimations). The baseline drift of the surrogate and target between scans was corrected. Various training periods for the baseline drift correction—i.e., correction periods (*T*_C_s)—were employed: *T*_C_ ∈ {5,10,15; *T*_C_ ≤ *T*_M_} [s]. Evaluations were conducted with and without the correction. The difference between the actual and estimated target positions was evaluated by the root-mean-square error (RMSE).

**Results:**

The range of mean respiratory period and 3D motion amplitude of the target was 2.4–13.0 s and 2.8–34.2 mm, respectively. On intra-scan estimation, the median 3D RMSE was within 1.5–2.1 mm, supported by previous studies. On inter-scan estimation, median elapsed time between scans was 10.1 min. All *T*_M_s exhibited 75th percentile 3D RMSEs of 5.0–6.4 mm due to baseline drift of the surrogate and the target. After the correction, those for each *T*_M_s fell by 1.4–2.3 mm. The median 3D RMSE for both the 10-s *T*_M_ and the *T*_C_ period was 2.4 mm, which plateaued when the two training periods exceeded 10 s.

**Conclusions:**

A widely-applicable estimation model for the 3D target positions during beam irradiation was developed. The optimal *T*_M_ and *T*_C_ for the model were both 10 s, to allow for more than one respiratory cycle.

**Trial registration:**

UMIN000014825. Registered: 11 August 2014.

**Electronic supplementary material:**

The online version of this article (10.1186/s13014-018-1019-9) contains supplementary material, which is available to authorized users.

## Background

Real-time detection of a target’s position in three-dimensional (3D) space is essential to ensure that high-dose radiation is delivered only to the target, where thus is possible with the aid of four-dimensional cone-beam computed tomography (4D-CBCT). However, the temporal resolution of 4D-CBCT is too low to precisely monitor target motion [1]. High-precision radiotherapy requires detection of the 3D position at high temporal resolution (sampling rate > 1 Hz). Localization of 3D target position during beam irradiation is used for the dose-of-the-day calculations [2], planning target volume verification [3], and real-time tumor tracking [4–6].

Optimal detection of time-resolved 3D position involves the triangulation of two simultaneous two-dimensional (2D) projections using pre-defined camera parameters [7]. Modern radiotherapy treatment machines are configured with a single-source kilovoltage (kV) imaging subsystem perpendicular to the megavoltage (MV) beam and an electronic portal imaging device. Some machines are equipped with orthogonal dual-source kV imaging subsystems; such machines allow kV/MV or kV/kV triangulation. However, depending on the extent of multi-leaf collimator motion during beam irradiation, the target on MV images may not be detectable. Detection of 3D target positions using a monoscopic view is now possible with techniques such as kV intra-fraction monitoring [8, 9] and marker-less tumor detection [10–12]. However, these methods are inapplicable if the target or tumor cannot be detected via kV projections at certain gantry angles.

To estimate target positions in these situations, there is no alternative but to employ external respiratory surrogates. Cho et al. developed a linear internal-external correlation model with frequent model updating during beam irradiation [13–15]. In their studies, the linear model was selected and updated by minimization of the difference between the measured kV projections and estimated trajectory projected onto a 2D projection geometry. As mentioned above, however, updating cannot be applied if the target or tumor cannot be detected on kV projections, as this would lead to model degradation. This problem has more of an impact on marker-less tumor tracking.

Akimoto et al. pointed out that the visibility of gold markers implanted in the lung on kV projections could be poor in the following situations: (1) overlap of the gold markers and (2) when there is a low-intensity ratio between the gold marker and its surroundings [16]. Bahig et al. investigated the applicability of a marker-less tumor tracking system using CyberKnife (XSight Lung Tracking System) via a fixed dual-source kV imaging subsystem [17]. They revealed that 50% of tumors smaller than 2 cm cannot be visualized on the kV projection. Thus, a means to evaluate the estimation model without updating during beam irradiation is required, regardless of whether gold markers are implanted.

Fassi et al. developed a target estimation method using a patient-specific breathing model derived a priori from 4D-CT images [18]. Model parameters were retrieved and updated for each treatment fraction according to in-room radiography acquisition and optical surface imaging. However, the low temporal resolution and imaging artifacts associated with 4D-CT may affect the model accuracy and cannot be improved by updating the model at each treatment fraction.

In this study, we developed a method to estimate target positions employing an external surrogate which was an infrared reflective (IR) marker; our approach is widely applicable and does not require additional imaging during beam irradiation. We applied the quadratic polynomial equation used by the Vero system [19]. The equation incorporated data from the internal target, and position and velocity data from the external IR marker placed on the patient’s abdomen; data were acquired at a frequency of 5 Hz. To validate the method, we used the dual-source 4D-CBCT projection data of lung cancer patients who underwent stereotactic body radiotherapy (SBRT); data included the actual 3D target positions during the 4D-CBCT scan, calculated from 2D orthogonal projections featuring kV/kV triangulation. First, the estimation equation was modeled using the detected 3D target positions and IR marker positions prior to target position estimation in the same scan (intra-scan estimation). Second, the equation was used to estimate target and surrogate positions in the validation session of another scan (inter-scan estimation). The baseline drifts of the target and IR marker between scans were corrected. Thus, the training periods for these sessions were optimized using the data from clinical practice.

## Methods

### Patient characteristics

Twenty-two consecutive lung cancer patients (17 males, 5 females; median age, 81 years; range: 65–90 years) who underwent SBRT by Vero4DRT (Mitsubishi Heavy Industries, Ltd., Hiroshima, Japan, and BrainLAB AG, Feldkirchen, Germany) after implantation of two-to-four 1.5-mm-diameter gold markers (Olympus, Tokyo, Japan) were enrolled in an institutional review board-approved trial. Each patient underwent three 70-s 4D-CBCT scans, except for Patient 17, and Patients 10 and 18, who underwent only one, and two scans, respectively. Three scans were performed for Patients 1 and 2 were employed in this study; the second scan of three scans for the other patients was taken for a different purpose, and with different scan parameters, and was not applied 4D-CBCT image reconstruction. Thus, the first and third scans of the other patients were used for the validation process. Both scans for Patients 10 and 18 were used for the validation.

A set of three IR markers was placed on each patient to record abdominal motion in the anterior-posterior (AP) direction. IR marker movement was captured using a portable Polaris Spectra camera (Northern Digital Inc., Ontario, Canada) placed close to the patient; the camera operated independently of the Vero4DRT system. Due to unexpected system problems, the IR marker signals from Patients 8 and 12 were not available. Thus, 20 patients were studied.

### Data acquisition

We recorded 351 projection images (70-s scans per source; i.e., the sampling time, Δ*t* = 0.2 s). All gold markers were simultaneously detected on all projections. The gold marker located nearest the tumor was considered the best surrogate of the target. The 2D positional data of each projection, or each discrete time *t*_*k*_ (*k* = 0, 1, ⋯, 350 [=70/Δ*t*]), were converted into 3D data using the pre-defined camera parameters, to yield actual 3D target positions *P*_actual_(*t*_*k*_). Data acquisition details have been described previously [1].

The respiratory period during a scan was defined as the average interval between two consecutive end inhalations. The respiratory amplitude was defined as the average difference between the end-exhalation and end-inhalation positions of a single respiratory cycle.

### Target position estimation using the external surrogate

**Fig. 1:**
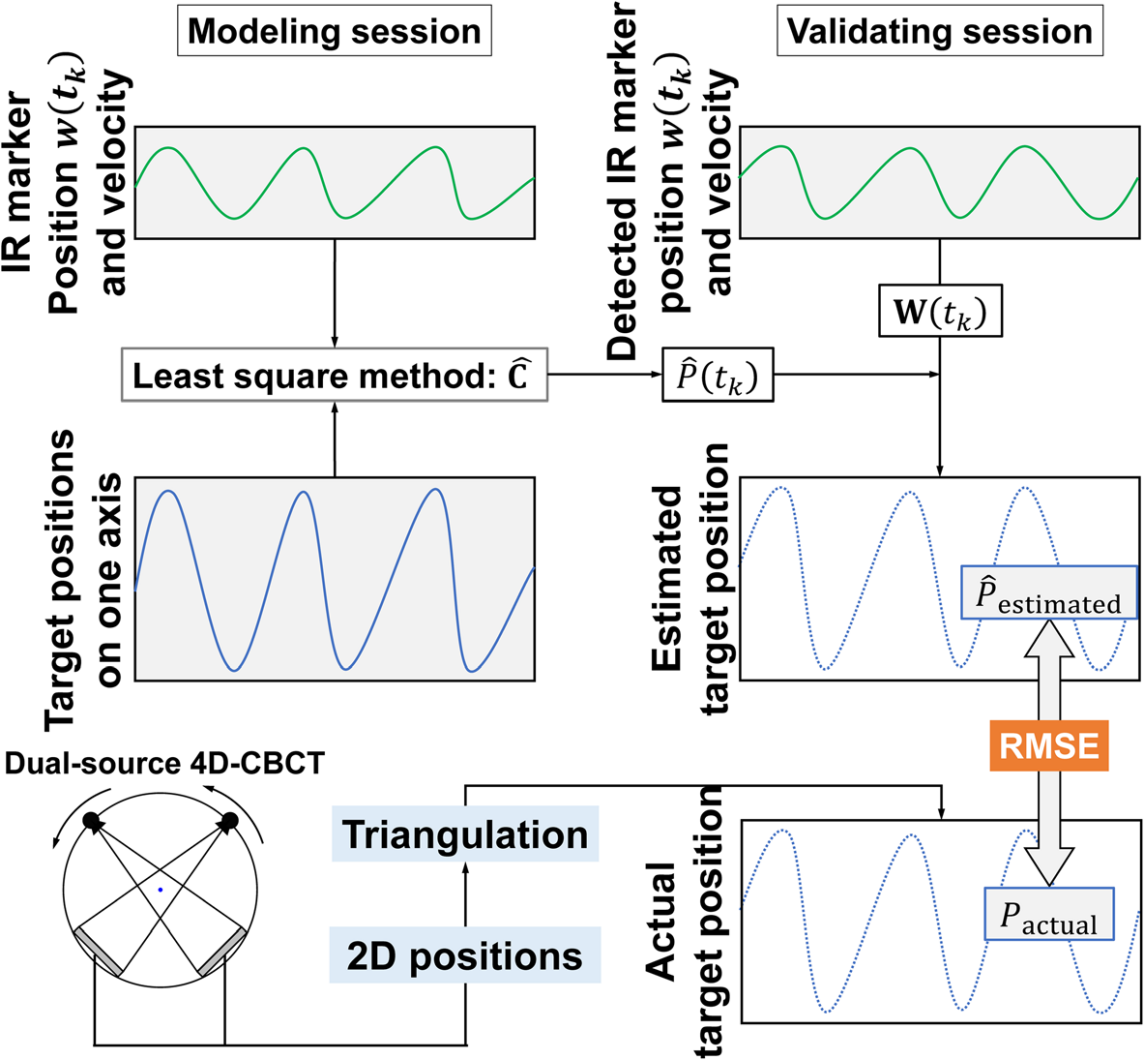
Flow and validation of our method. The estimation equation, *P*(*t*_*k*_), is constructed during the modeling session. *t*_*k*_ is the discrete time. The root-mean-square error (RMSE) between the estimated target position, ${\widehat{P}}_{\mathrm{estimated}}$, and the actual target position, *P*_actual_, is calculated during the validation session. **C**: coefficients for *P*(*t*_*k*_). **W**(*t*_*k*_): the position and velocity of the infrared reflective (IR) marker at *t*_*k*_. 2D: two-dimensional

Our proposed estimation method is shown in Fig. 1. We assumed that the target position would be estimated during beam irradiation using an estimation equation modeled before the irradiation. Thus, we considered two sessions: the modeling and validating sessions. To estimate the target positions during the validating session, construction of the estimation equation was necessary during the modeling session. The quadratic polynomial equation used in the proposed method can be written as follows:

1 $$ {P}_{i,\gamma}\left({t}_k\right)={a}_{i,\gamma }{w}^2\left({t}_k\right)+{b}_{i,\gamma }w\left({t}_k\right)+{c}_{i,\gamma }+{d}_{i,\gamma }{\left(\frac{\Delta w}{\Delta t}\right)}^2+{e}_{i,\gamma}\left(\frac{\Delta w}{\Delta t}\right)\equiv {\mathbf{C}}_{i,\gamma}\bullet \mathbf{W}\left({t}_k\right). $$

**C**_*i*, *γ*_ = (*a*_*i*, *γ*_ *b*_*i*, *γ*_ *c*_*i*, *γ*_ *d*_*i*, *γ*_ *e*_*i*, *γ*_) is the coefficient of the equation for IR marker number *i* ∈ {1, 2, 3} in direction *γ* ∈ {left − right (LR), superior − inferior (SI), anterior − posterior}, and $\mathbf{W}\left({t}_k\right)={\left({w}^2\left({t}_k\right)\kern0.5em w\left({t}_k\right)\kern0.5em 1\kern0.5em {\left(\frac{\Delta w}{\Delta t}\right)}^2\kern0.5em \left(\frac{\Delta w}{\Delta t}\right)\right)}^{\mathrm{T}}$ is the IR marker data set at the discrete time *t*_*k*_, containing the IR marker position *w*(*t*_*k*_) and its derivative—i.e., velocity, Δ*w*/Δ*t*—where Δ*w* = *w*(*t*_*k*_) − *w*(*t*_*k* − 1_) andΔ*t* = *t*_*k*_ − *t*_*k* − 1_ = 0.2 [s]. Because the following methodology is the same for any IR marker number and direction, we eliminated *i* and *γ* for simplicity. We divided each of the 351 projections into a first (175 projections, approximately 35 s) and second (176 projections, approximately 35 s) half for modeling and validating sessions, respectively.

**Fig. 2:**
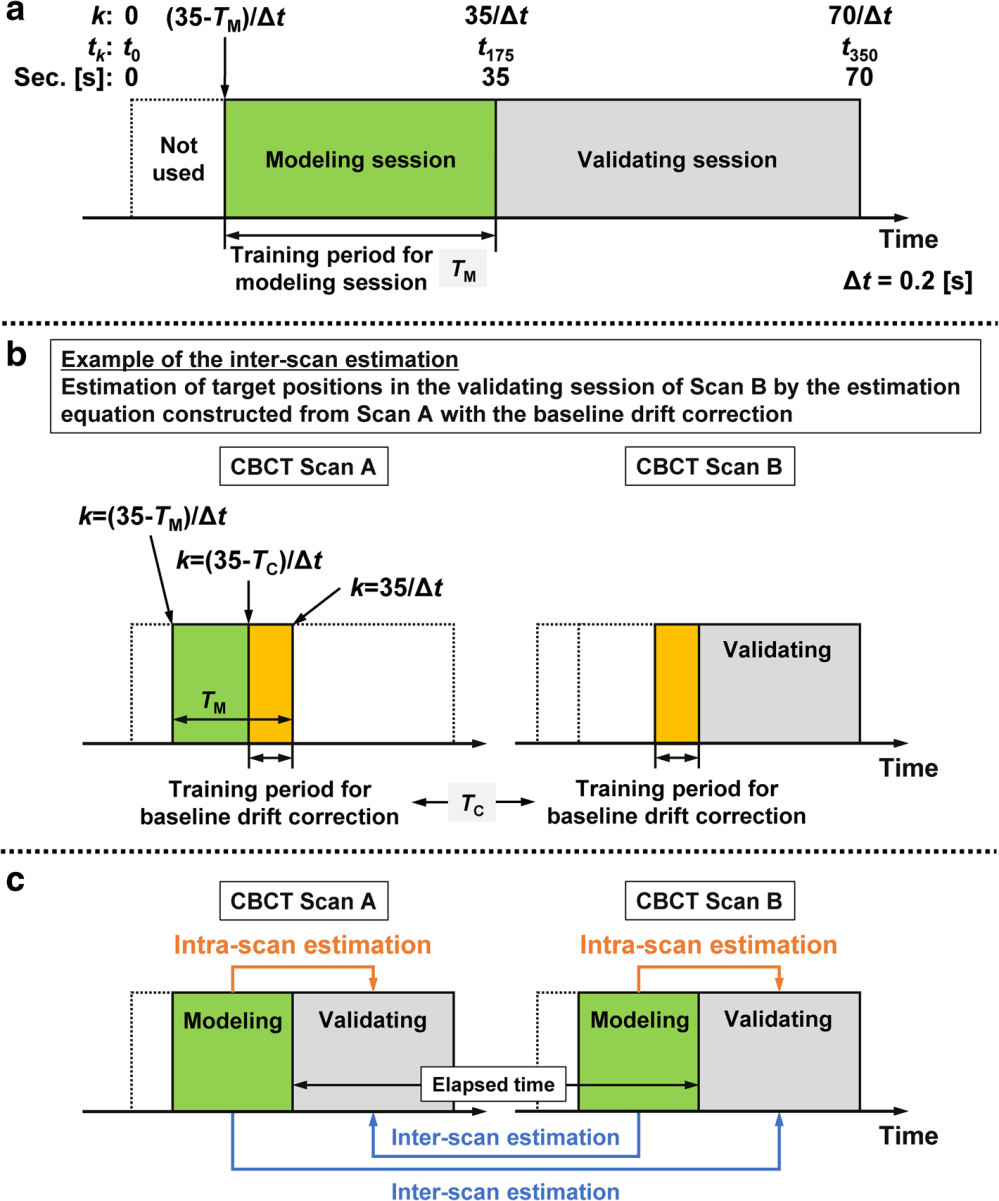
**a** Details of the training period for the modeling session (*T*_M_); Δ*t* is the sampling rate. **b** Example of an inter-scan estimation. The relationship between *T*_M_ and the training period for the baseline drift correction (*T*_C_) is shown. ${\left[{\widehat{P}}_{\left({T}_{\mathrm{M}},{T}_{\mathrm{C}}\right)}^{\mathrm{A}\to \mathrm{B}}\left({t}_k\right)\right]}^{\mathrm{C}\mathrm{or}}$ is the baseline drift-corrected estimation equation. **c** The relationship between intra- and inter-scan estimations on two cone-beam computed tomography (CBCT) scans

First, we determined the coefficients of the estimation equation during the modeling session and then estimated the target positions during the validating session of the same scan; i.e., an intra-scan estimation. To construct the estimation equation, coefficient **C** was determined by the least-squares method using the data of the modeling session. We employed various training periods for modeling—i.e., the modeling period *T*_M_ ∈ {5,10,15,25,35} [s]—as shown in Fig. 2a. Thus, a coefficient was determined for each *T*_M_ and written as:

2 $$ {\widehat{\mathbf{C}}}_{T_{\mathrm{M}}}=\mathrm{argmin}\sum \limits_{k=\left(35-{T}_{\mathrm{M}}\right)/\Delta t+1}^{35/\Delta t}{\left\Vert {P}_{\mathrm{actual}}\left({t}_k\right)-\mathbf{C}\bullet \mathbf{W}\left({t}_k\right)\right\Vert}^2. $$

Note that the data at ${t}_{\left(35-{T}_{\mathrm{M}}\right)/\Delta t}$ were also used because the velocity of the IR marker was necessary. Hereinafter, we use the hat symbol for coefficients determined by the least-squares method, and the estimation equation that included the coefficients, or ${\widehat{P}}_{T_{\mathrm{M}}}\left({t}_k\right)$. The target positions within the validating session of the same scan were estimated using the estimation equation in each direction.

Second, we estimated the target positions in the validating session using the estimation equation constructed during the modeling session of another scan in a one patient; i.e., an inter-scan estimation (Fig. 2b). In clinical cases, the baseline drift correction will be applied between fields or arcs if multiple beams are used in the plan. Let ${\widehat{P}}_{T_{\mathrm{M}}}^{\mathrm{A}\to \mathrm{B}}\left({t}_k\right)$ be the estimation equation for the target positions of scan B by using the coefficients determined by the data of scan A. Due to the elapsed time between the two scans, the baseline drift of the IR marker and target should be corrected. The baseline of scan A is defined as the median position in the training period for the baseline drift correction—i.e., the correction period *T*_C_ ∈ {5,10,15; *T*_C_ ≤ *T*_M_} [s]—at the end of the modeling session or prior to the validating session (Fig. 2b). The baseline of scan B is defined as the median position in the correction period *T*_C_ immediately prior to the validating session of scan B. The amount of the baseline drift between scans A and B was calculated by subtracting the baseline of scan A from that of scan B. First, we corrected the baseline drift of the IR marker. The amount of baseline drift of the IR marker, defined as follows:

3 $$ {\mathrm{B}\mathrm{D}}_{\mathrm{IR},{T}_{\mathrm{C}}}^{\mathrm{B}-\mathrm{A}}=\mathrm{median}\left\{{w}^{\mathrm{B}}\left({t}_k\right),k\in \left\{\frac{35-{T}_{\mathrm{C}}}{\Delta t}:\frac{35}{\Delta t}\right\}\right\}-\mathrm{median}\left\{{w}^{\mathrm{A}}\left({t}_k\right),k\in \left\{\frac{35-{T}_{\mathrm{C}}}{\Delta t}:\frac{35}{\Delta t}\right\}\right\}. $$

Then, we incorporated the baseline drift of IR marker by transforming *w*^B^(*t*_*k*_) into ${w}^{\mathrm{B}}\left({t}_k\right)-{\mathrm{B}\mathrm{D}}_{\mathrm{IR},{T}_{\mathrm{C}}}^{\mathrm{B}-\mathrm{A}}$ and let ${\left[{\widehat{P}}_{\left({T}_{\mathrm{M}},{T}_{\mathrm{C}}\right)}^{\mathrm{A}\to \mathrm{B}}\left({t}_k\right)\right]}^{\prime }$ be the estimation equation ${\widehat{P}}_{T_{\mathrm{M}}}^{\mathrm{A}\to \mathrm{B}}\left({t}_k\right)$ scaled by ${\mathrm{B}\mathrm{D}}_{\mathrm{IR},{T}_{\mathrm{C}}}^{\mathrm{B}-\mathrm{A}}$. To incorporate the baseline drift of the target, we compensated for internal residual errors resulting from the baseline drift of ${\left[{\widehat{P}}_{T_{\mathrm{M}}}^{\mathrm{A}\to \mathrm{B}}\left({t}_k\right)\right]}^{\prime }$—i.e., ${\mathrm{B}\mathrm{D}}_{\mathrm{Target},{T}_{\mathrm{C}}}^{\mathrm{B}-\mathrm{A}}$—which can be written as

4 $$ {\mathrm{B}\mathrm{D}}_{\mathrm{Target},{T}_{\mathrm{C}}}^{\mathrm{B}-\mathrm{A}}=\mathrm{median}\left\{{\left[{\widehat{P}}_{\left({T}_{\mathrm{M}},{T}_{\mathrm{C}}\right)}^{\mathrm{A}\to \mathrm{B}}\left({t}_k\right)\right]}^{\prime },k\in \left\{\frac{35-{T}_{\mathrm{C}}}{\Delta t}:\frac{35}{\Delta t}\right\}\right\}-\mathrm{median}\left\{{P}_{\mathrm{actual}}^{\mathrm{A}}\left({t}_k\right),k\in \left\{\frac{35-{T}_{\mathrm{C}}}{\Delta t}:\frac{35}{\Delta t}\right\}\right\}. $$

Then, we subtracted ${\mathrm{B}\mathrm{D}}_{\mathrm{Target},{T}_{\mathrm{C}}}^{\mathrm{B}-\mathrm{A}}$ from ${\left[{\widehat{P}}_{\left({T}_{\mathrm{M}},{T}_{\mathrm{C}}\right)}^{\mathrm{A}\to \mathrm{B}}\left({t}_k\right)\right]}^{\prime }$, and let ${\left[{\widehat{P}}_{\left({T}_{\mathrm{M}},{T}_{\mathrm{C}}\right)}^{\mathrm{A}\to \mathrm{B}}\left({t}_k\right)\right]}^{\mathrm{C}\mathrm{or}}$ be the baseline drift-corrected estimation equation. To increase the number of data sets, we estimated the target positions in the validating session of scan A using the estimation equation constructed in the modeling session of scan B. The relationship between the intra- and inter-scan estimations is shown in Fig. 2c. The 4D-CBCT projection data for Patient 17 were only used to validate the intra-scan estimation, because the patient underwent 4D-CBCT only once.

For comparison, we also used a *T*_M_ of 70 s. Note that the validating session was included to model the estimation equation ${\widehat{P}}_{T_{\mathrm{M}}=70}\left({t}_k\right)$ for the intra-scan estimation. For the inter-scan estimation, the baseline drifts of the IR marker and target were calculated, and correction was carried out using the process described above.

The linear estimation equation constructed from eight 4D-CBCT positions was also utilized. Eight averaged target positions for each phase, *P*_4D − CBCT_(*l*) (*l* ∈ {0, 1, ⋯, 7}), were obtained from one 4D-CBCT scan. In addition, eight averaged IR marker positions, *w*_ave_(*l*) (*l* ∈ {0, 1, ⋯, 7}), were obtained for each phase. We defined phases 0 and 4 as end-exhalation and end-inhalation, respectively. The linear estimation equation was constructed between each phase; between phase *α* and *β,* it was written as follows:

$$ {\widehat{P}}_{\mathrm{Linear}}^{\left(\alpha, \beta \right)}\left({t}_k\right)={a}^{\left(\alpha, \beta \right)}w\left({t}_k\right)+{b}^{\left(\alpha, \beta \right)} $$

5 $$ \mathrm{if}\ {w}_{\mathrm{ave}}\left(\alpha \right)\le w\left({t}_k\right)\le {w}_{\mathrm{ave}}\left(\beta \right),\left(\alpha, \beta \right)\in \left\{\left(0,1\right),\left(1,2\right),\cdots, \left(7,0\right)\right\} $$

In the validating session, the IR marker position *w*(*t*_*k*_) was distinguished as mid-exhalation and mid-inhalation when Δ*w*/Δ*t* > 0 and Δ*w*/Δ*t* < 0, respectively. In the inter-scan estimation, the centroid position of the eight averaged target positions was recognized as the baseline for a 4D-CBCT scan. The baseline drift correction for Eq. (5) was employed similarly as mentioned above. The number of internal residual errors resulting from the baseline drift of ${\left[{\widehat{P}}_{\mathrm{Linear}}^{\left(\alpha, \beta \right)}\left({t}_k\right)\right]}^{\prime }$ is given by

6 $$ {\mathrm{B}\mathrm{D}}_{\mathrm{Target},\left({T}_{\mathrm{C},4\mathrm{D}-\mathrm{CBCT}}\right)}^{\mathrm{B}-\mathrm{A}}=\mathrm{median}\left\{{\left[{\widehat{P}}_{\mathrm{Linear}}^{\left(\alpha, \beta \right)}\left({t}_k\right)\right]}^{\prime },k\in \left\{\frac{35-{T}_{\mathrm{C}}}{\Delta t}:\frac{35}{\Delta t}\right\}\right\}-\frac{1}{8}\sum \limits_{l=0}^7{P}_{4\mathrm{D}-\mathrm{CBCT}}^{\mathrm{A}}(l). $$

Because the estimation equation is constructed for each IR marker *i*, the estimated target position is defined as the average of the target position estimated from each IR marker, written as follows: ${\widehat{P}}_{\mathrm{estimated}}\left({t}_k\right)=\frac{1}{3}\sum \limits_{i=1}^3{\widehat{P}}_i\left({t}_k\right)$. The difference between the estimated target position ${\widehat{P}}_{\mathrm{estimated}}\left({t}_k\right)$ and actual target position *P*_actual_(*t*_*k*_) in each direction was evaluated in terms of the root-mean-square error (RMSE).

### Statistical analysis

We tested for equality of variance prior to performing multiple pairwise comparisons. Depending on the equality of variance status, we used either one-way analysis of variance or the non-parametric Kruskal–Wallis test to compare the differences. If a difference was significant, the non-parametric Steel–Dwass test was performed subsequently to simultaneously evaluate all differences between training periods. A *p*-value < 0.05 was considered statistically significant.

## Results

### Respiratory patterns

**Fig. 3:**
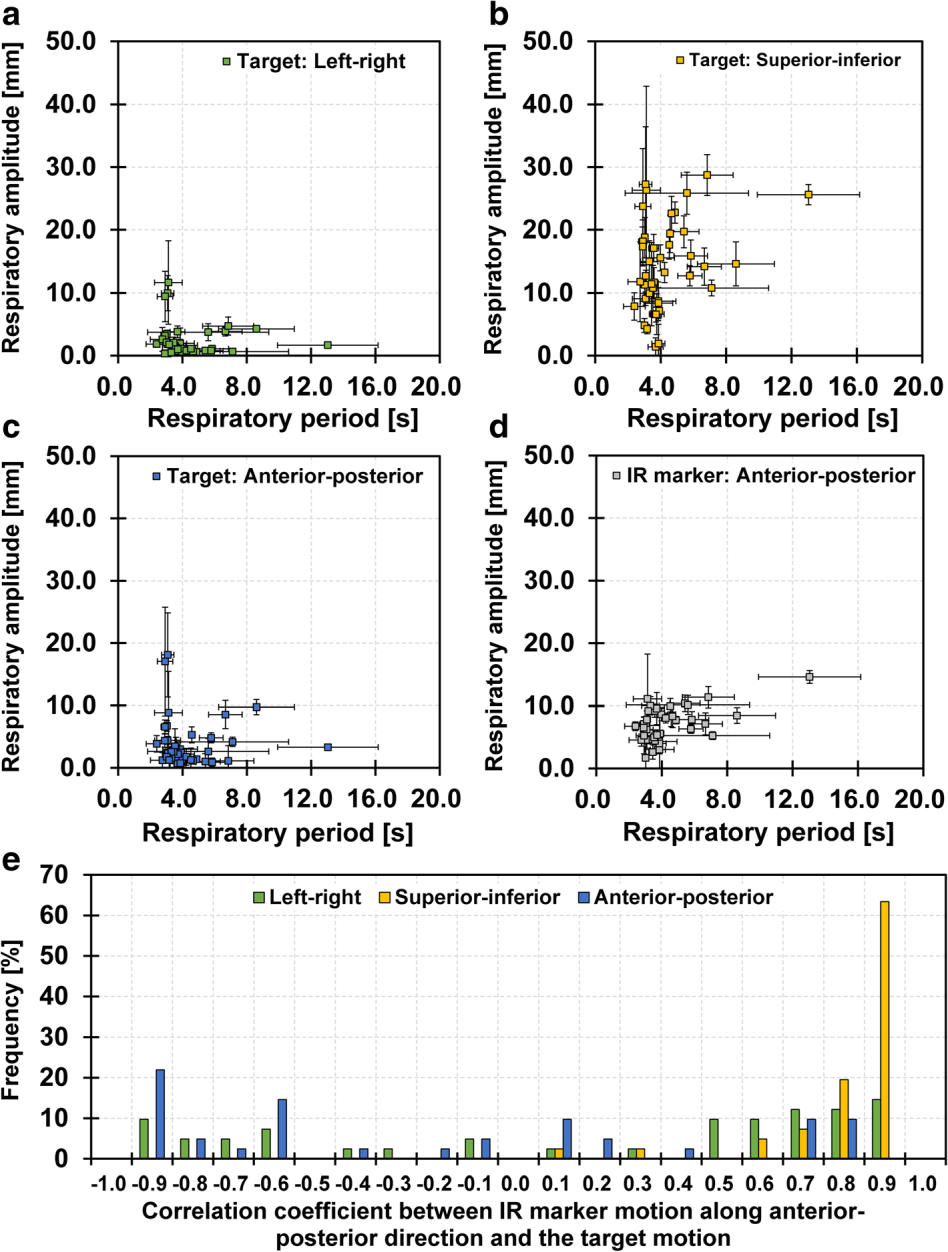
Scatter plots of the respiratory periods of all patients, and the target motion amplitude in (**a**) the left-right, (**b**) the superior-inferior, and (**c**) the anterior-posterior (AP) directions; and (**d**) that of the infrared reflective (IR) marker in the AP direction. (**e**) Correlation coefficients between IR marker motion in the AP direction and target motion

The range of mean period was 2.4–13.0 s; the range of mean IR marker amplitude in the AP direction was 1.7–14.6 mm; and the range of mean target amplitude in the LR, SI, AP directions, and 3D motion were 0.3–11.7, 1.4–28.7, 0.7–18.1, and 2.8–34.2 mm, respectively. The respiratory amplitudes during the various scan periods of all patients, and the correlation coefficients between IR marker motion along the AP direction and target motions along the LR, SI, and AP directions, are shown in Fig. 3; large error bars indicate irregular breathing, such as apnea, tachypnea, hypopnea, hyperpnea, or combinations of these. The target motion in the SI direction was strongly positively correlated with IR marker motion.

### Intra-scan estimations

**Fig. 4:**
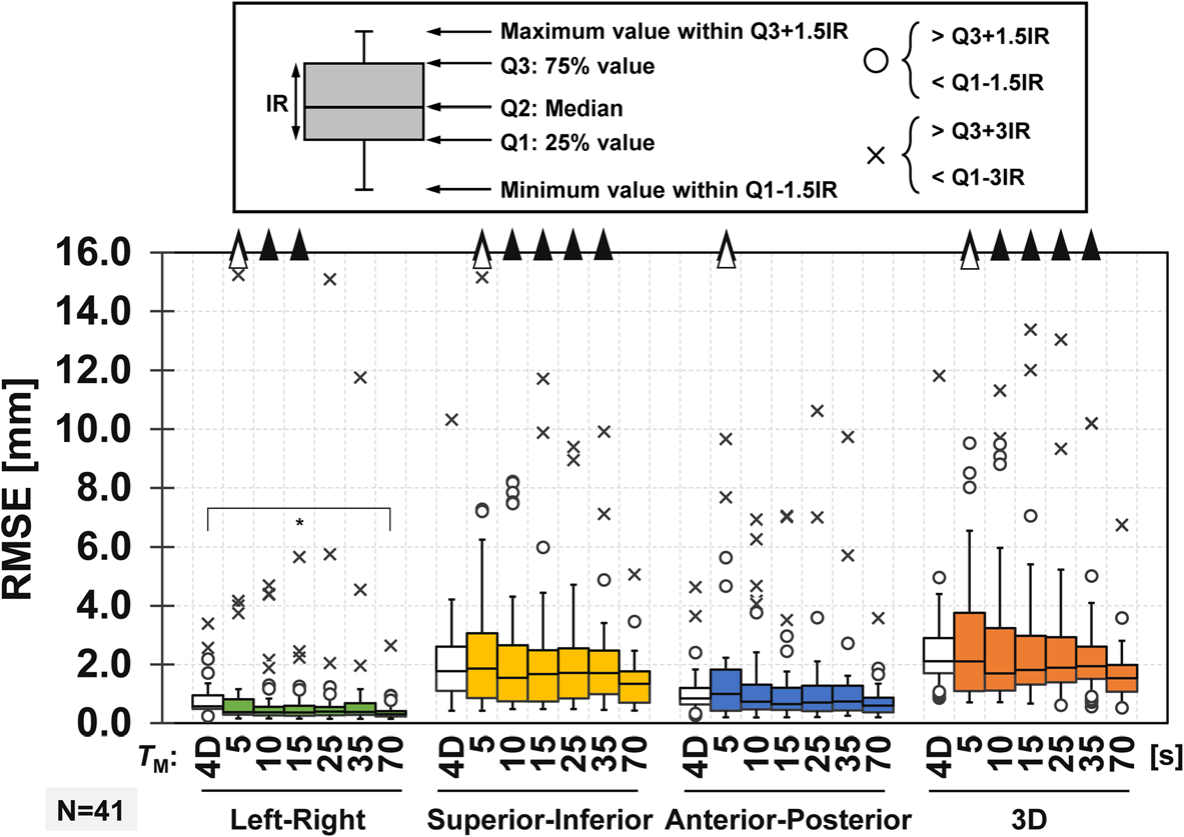
Boxplots of RMSEs for each training period within a modeling session (the *T*_M_ values) in the left-right, superior-inferior, anterior-posterior, and three-dimensional (3D) directions, calculated via intra-scan estimations. White triangles: outliers out of range. Black triangles: outliers observed in patient included apnea and hyperpnea data. *p < 0.05

The RMSEs in the LR, SI, AP, and 3D directions of the intra-scan estimations are shown in Fig. 4. The RMSEs in the SI direction were associated with larger errors than those of the other directions. The median RMSEs in the LR and AP directions were < 1.0 mm. The median 3D RMSEs for all *T*_M_s were 1.5–2.1 mm and gradually declined as *T*_M_ became longer. Although the Kruskal–Wallis test revealed significant differences among *T*_M_s values in the LR direction (*p* < 0.05), the 3D RMSEs did not differ significantly. However, the *T*_M_s of 5 s—i.e., *T*_M_ = 5 s—showed large outliers in all directions, and the 3D RMSE of Patient 10 resulted in large outliers in *T*_M_ = 5, 10, 15, 25, and 35 s. Note that although the estimation equation modeled by *T*_M_ = 4D-CBCT and *T*_M_ = 70 s showed small errors, these *T*_M_s had included *P*_actual_ to construct the linear and quadratic estimation equations, respectively.

### Inter-scan estimations

**Fig. 5:**
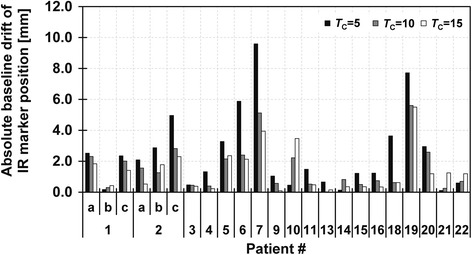
Absolute baseline drift of the IR marker, which calculated during each baseline drift correction period (*T*_C_ = 5, 10, and 15 s). In the graph, a, b, and c for Patients 1 and 2 indicate the baseline drift between first and second scans, first and third scans, and second and third scans, respectively

**Fig. 6:**
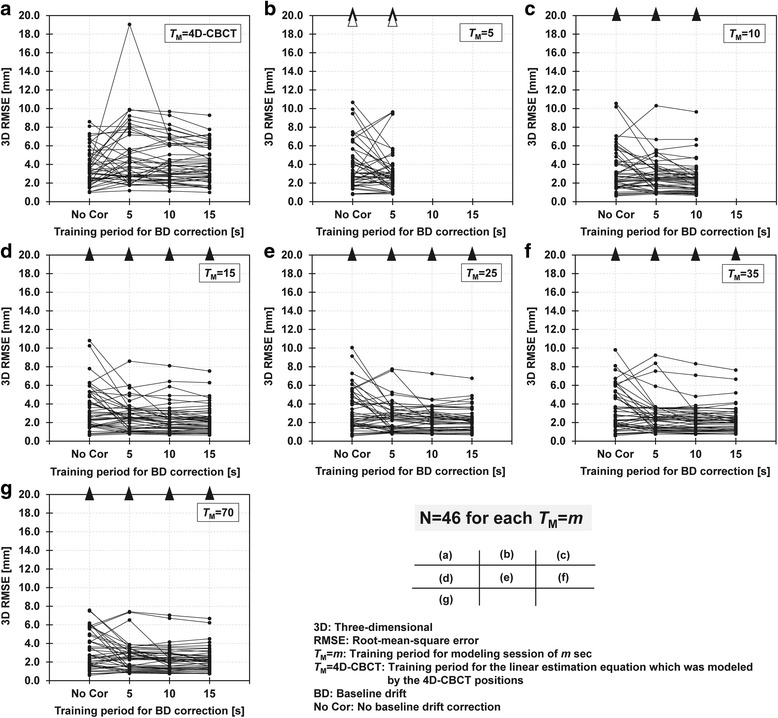
Changes in 3D RMSEs due to differences in the correction period for the modeling period of (**a**) four-dimensional cone-beam computed tomography, (**b**) 5 s, (**c**) 10 s, (**d**) 15 s, (**e**) 25 s, (**f**) 35 s, and (**g**) 70 s. White triangles: outliers out of range. Black triangles: outliers observed in patient included apnea and hyperpnea data

The median elapsed time between scans was 10.1 min (range: 1.7–15.0 min). The absolute baseline drift of an IR marker, which was calculated during each baseline drift *T*_C_ is shown in Fig. 5. The median absolute baseline drift for *T*_C_ = 5, 10, and 15 s was 1.5 mm (range: 0.1–9.6 mm), 0.8 mm (range: 0.0–5.0 mm), and 1.2 mm (range: 0.0–5.5 mm), respectively. No significant difference among each *T*_C_ values was found. The time series of the 3D RMSEs in *T*_C_ for each *T*_M_ is shown in Fig. 6. Although 3D RMSEs for all *T*_M_s exhibited large values without the baseline drift correction, they were decreased drastically by the correction, except for the 4D-CBCT data.

**Fig. 7:**
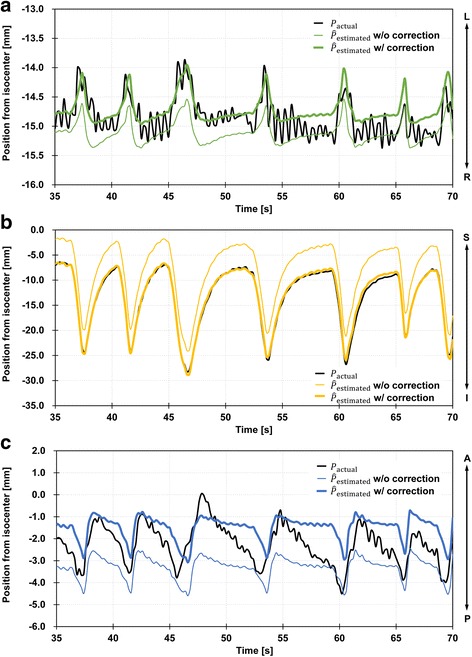
Actual (*P*_actual_) and estimated target positions (${\widehat{P}}_{\mathrm{estimated}}$) in the (**a**) left-right (LR), (**b**) superior-inferior (SI), and (**c**) anterior-posterior (AP) directions with and without the baseline drift correction of the first scan of Patient 2, estimated from the estimation equation modeled by the data of the third scan. Both the modeling and correction periods was 10 s

Figure 7 shows *P*_actual_ and ${\widehat{P}}_{\mathrm{estimated}}$ without and with baseline drift correction of the first scan of Patient 2, determined using the estimation equations ${\widehat{P}}_{T_{\mathrm{M}}=10}^{3\mathrm{rd}\to 1\mathrm{st}}\left({t}_k\right)$ and ${\left[{\widehat{P}}_{\left({T}_{\mathrm{M}}=10,{T}_{\mathrm{C}}=10\right)}^{3\mathrm{rd}\to 1\mathrm{st}}\left({t}_k\right)\right]}^{\mathrm{C}\mathrm{or}}$, respectively; both the *T*_M_ and the *T*_C_ was 10 s. In this case, 3D RMSE was reduced from 5.4 mm to 1.1 mm with the baseline drift correction. In contrast, in several cases the 3D RMSE was over 6 mm even with the baseline drift corrections, due to a sudden change in the correlation between the IR marker and the target in the validating session in Fig. 6. Once again, the *T*_M_ = 5 s data exhibited large outliers in all directions. Moreover, the 3D RMSE of Patient 10 resulted in severe outliers in all *T*_M_s, except for the *T*_M_ = 4D-CBCT data.

**Fig. 8:**
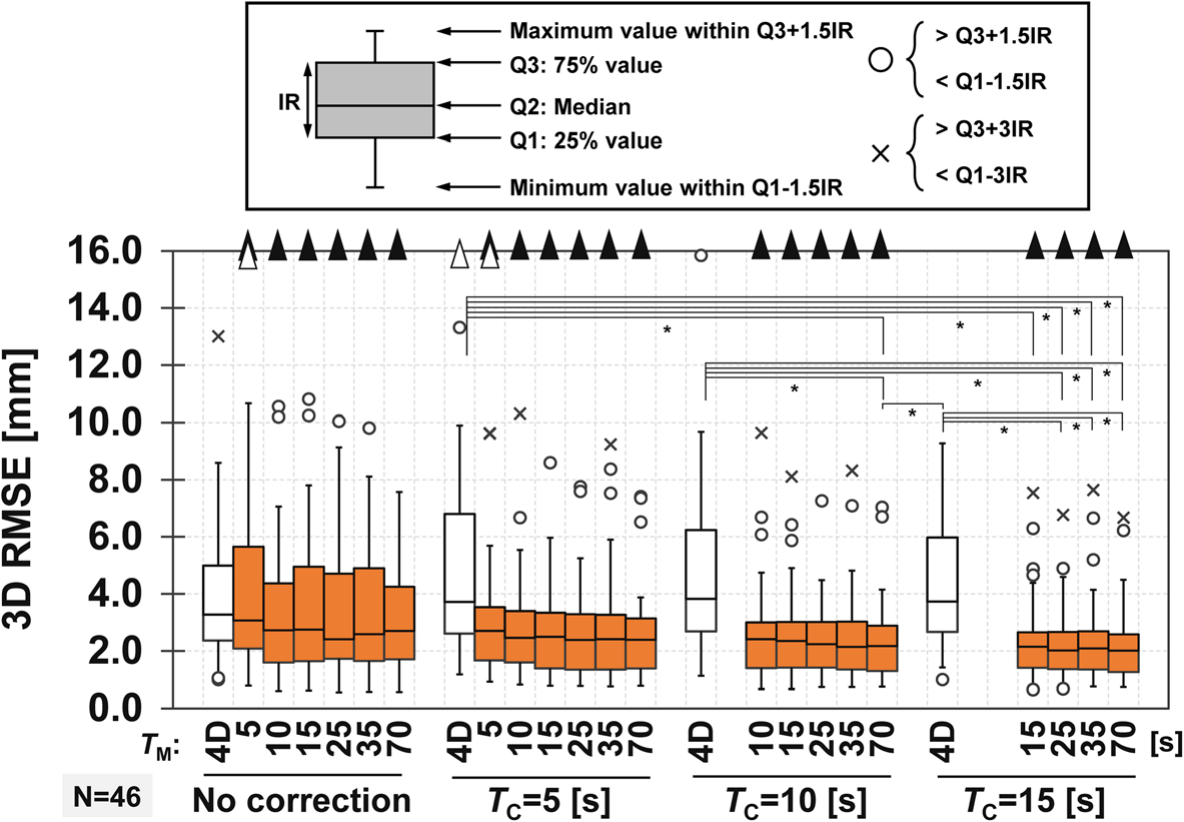
Boxplots of the 3D RMSEs for each training period in terms of the modeling session (*T*_M_) and baseline drift correction (*T*_C_). White triangles: outliers out of range. Black triangles: outliers observed in patient included apnea and hyperpnea data. *p < 0.05

Boxplots of the 3D RMSEs are shown in Fig. 8. The 75th percentile 3D RMSEs without baseline drift correction were 5.0–6.4 mm. After baseline drift correction, the 75th percentile 3D RMSEs for each *T*_M_s fell by 1.4–2.3 mm, except for the *T*_M_ = 4D-CBCT, which increased by 0.6–1.2 mm. Prolongation of both *T*_M_ and *T*_C_ reduced the median 3D RSMEs; however, this effect plateaued at *T*_M_s and *T*_C_s > 10 s. The Steel–Dwass test revealed significant differences between *T*_M_ = 4D-CBCT with baseline drift correction and several *T*_M_s (all *p* < 0.05).

## Discussion

Similar to other studies [20, 21], the patients enrolled in this study exhibited various combinations of respiratory period and amplitude; the mean respiratory period was approximately 4 s. Target motion along SI direction had strong correlation with IR marker motion along the AP direction, as also found by Ionascu et al. [22]. Several target motions correlated only weakly with IR marker motion because breathing was sometimes irregular, as shown by the large error bars on the amplitude axis in Fig. 3a–3d. As target positions were estimated using only the IR marker, the weak correlations between target and IR marker positions created large RMSEs [23].

Following intra-scan estimations, the median RMSEs for the estimated target positions—i.e., —in the LR, AP, and SI directions were 1-2 mm, as also found by Ruan et al. [24]. Although the 75th percentile 3D RMSE for the *T*_M_ of 5 s was < 4 mm in most patients, several large RMSEs were observed. The proportion of patients with a respiratory period within 5 and 10 s was 78 and 98%, respectively. Thus, *T*_M_ must cover at least one respiratory cycle, which was set to 10 s in this study. The median 3D RMSEs fell as the *T*_M_ was prolonged. The median 3D RMSE for *T*_M_ = 10 s and *T*_M_ = 35 s was 2.1 and 2.0 mm, respectively. No significant difference was apparent among the RMSEs for each *T*_M_. Therefore, a training period of 10 s would be appropriate to model the estimation equation. In comparison, *T*_M_ = 70 showed a median 3D RMSE of 1.7 mm.

Cho et al. developed a method in which the 3D target position could be estimated using a linear equation that included the position of the external surrogate, and thus requires frequent model updating was required during beam irradiation [13]. Forty-six thoracic and abdominal cancer patients were investigated in their study and they calculated that updates at 1 Hz yielded 3D RMSEs of nearly 1 mm, similar to the RMSEs of stereoscopic estimations. However, the range in the mean 3D motion of the targets located at the left or right lower lobe of the lungs was 0.2–14.4 mm in the study [13–15, 25], whereas in our study we observed a range of 3.7–28.4 mm. For instance, excluding the data that showed a 3D target amplitude larger than 14.4 mm, the 3D RMSE for *T*_M_ = 10 s was 1.1 mm (data shown in Additional file 1). As mentioned above, updating is impossible if the kV image is temporarily unavailable, which would lead to degradation of the model and greater impact on marker-less tumor tracking. Again, the implanted gold markers were not always visible in situations with marker overlap and low marker contrast relative to the surroundings. Moreover, the model developed by Fassi et al. required no additional imaging during beam irradiation [18]. Although the cited authors evaluated the estimation errors from 2D positions on CBCT, which were projected from estimated 3D positions, the errors were about 2 mm. One advantage of our method is that MV and kV images are not required during beam irradiation; additionally, the RMSE associated with our method is comparable to those of previous study [13].

Regarding inter-scan estimations, the estimation equations were applied to other scans, and optimal *T*_M_ and *T*_C_ values were determined. The 3D RMSEs for the *T*_C_ of each *T*_M_ fell when the baseline drift corrections were applied, except in the case of *T*_M_ = 4D-CBCT. Twenty-nine of forty-six inter-scan estimations for *T*_M_ = 4D-CBCT estimations exhibited no improvement upon baseline drift correction. The 3D RMSEs for corrected *T*_M_ = 4D-CBCT values showed a larger interquartile range and longer error bars than did those without the correction, as well as the 3D RMSEs of all other *T*_M_s (Fig. 8). This is because the power of the linear estimation equation is first-order in nature, and thus is insensitive to baseline drift correction. On the other hand, the 3D RMSEs of other *T*_M_s after correction exhibited small interquartile ranges and lower error bars. The median 3D RMSE fell as *T*_M_ and *T*_C_ were prolonged, plateauing at *T*_M_ = 10 s for each *T*_C_. The minimum median 3D RMSE was 2.0 mm when *T*_M_ = 70 s and *T*_C_ = 15 s were combined; the difference between this value and the median 3D RMSE of the *T*_M_ = 10 s plus *T*_C_ = 10 s combination was < 0.4 mm. No significant differences were apparent among the RMSEs for *T*_M_ = 70 or the other *T*_M_s, except in the case of *T*_M_ = 4D-CBCT. Based on the population of patients and results of the inter-scan estimation, a combination of *T*_M_ = 10 s and *T*_C_ = 10 s would be appropriate.

Our method is independent of the beam delivery technique and is widely applicable. Although no additional imaging is needed during beam irradiation, baseline drift correction, applied between fields or arcs, may be required in some cases. Thomas et al. investigated treatment times, including the time from the start of the first field or arc to the cessation of the last [26]. The treatment time of patients undergoing lung SBRT via flattening filter-associated intensity-modulated radiotherapy was ~ 15 min. In such cases, baseline drift correction may be essential; the median elapsed time in this study was 10.1 min. However, during flattening filter-free volumetric-modulated arc therapy, the treatment time can be reduced to < 3.3 min. Thus, baseline drift correction may not be necessary with a short treatment time.

One limitation of this study was the additional imaging dose. The imaging dose delivered to a 2-cm^3^ volume of skin during a 70-s dual-source 4D-CBCT scan was 7.4–10.5 cGy [27]. The imaging dose delivered by our method during the 10-s training period was approximately one-seventh of that value. However, if abdominal motion is captured during 3D-CBCT performed to ensure appropriate patient positioning, projection data can also be used to model the estimation equation; thus, in the absence of baseline drift correction, no additional imaging doses would be required.

## Conclusions

We developed a widely applicable method to estimate 3D target positions using an external respiratory surrogate without additional imaging during beam irradiation, and optimized training periods for modeling and baseline drift correction by reference to clinical data. The developed method is independent of beam delivery technique. We revealed that a long training period was not necessary; the optimal training period to model the estimation equation and baseline drift correction was determined to be 10 s, to include more than one respiratory cycle for most patients in this study.

## Additional file

Additional file 1: Dataset supporting our findings. (XLSX 1259 kb)

## Abbreviations

2D: Two-dimensional
3D: Three-dimensional
4D: Four-dimensional
AP: Anterior-posterior
CBCT: Cone-beam computed tomography
CT: Computed tomography
IR: Infrared reflective
kV: Kilovoltage
LR: Left-right
MV: Megavoltage
RMSE: Root-mean-square error
SBRT: Stereotactic body radiotherapy
SI: Superior-inferior
